# The Level of Stress and Anxiety in Pregnant Women Depending on Social Support and Physical Activity

**DOI:** 10.3390/jcm12093143

**Published:** 2023-04-27

**Authors:** Joanna Kowalska

**Affiliations:** Faculty of of Physiotherapy, Wroclaw University of Health and Sport Sciences, 51-612 Wroclaw, Poland; joanna.kowalska@awf.wroc.pl

**Keywords:** stress, anxiety, physical activity, social support, pregnant women, emotional state

## Abstract

Social support and physical activity are mentioned among the numerous factors affecting the emotional state of pregnant women. Therefore, the aim of the study was to assess the level of perceived stress and anxiety in pregnant women depending on social support and their physical activity both before and during pregnancy and find the factors that affected the level of perceived stress. Methods. A total of 373 pregnant women were qualified for the study. The Perceived Stress Scale (PSS-10), the State-Trait Anxiety Inventory (STAI), and a self-administered questionnaire were used. Results. In the study group, a high level of stress and anxiety were noted. A comparative analysis showed that physically active women before pregnancy, women attending childbirth classes and remaining in a relationship, were characterized by a lower level of stress and anxiety compared to physically inactive women, women who did not participate in childbirth classes and were single. A multiple linear regression analysis showed that participation in childbirth classes, physical activity before pregnancy, the level of anxiety as a trait, and women’s age had the most significant impact on the stress level of surveyed women. Conclusions. Further research among pregnant women and women in the postpartum period is needed to confirm the benefits of physical activity and to identify as many factors as possible that may affect the emotional state of pregnant women.

## 1. Introduction

Pregnancy is a crucial period in women’s lives, during which they experience biological and psychological changes as well as changes in their status in the family and society. These changes can lead to mental disorders of different types. The most common include increased levels of perceived stress and anxiety, and mood disorders [1,2,3]. These prenatal mental disorders are associated with undesirable effects for the mother and fetus. They may contribute to complications during pregnancy and childbirth and affect the child’s health and development [4,5,6,7]. Additionally, there is an increased risk of postnatal depression in women experiencing prenatal mental disorders [8,9].

Previous research indicates that the lack of social support is mentioned among numerous factors negatively affecting the emotional state of pregnant women [1,2,10]. Research by Li et al. indicates that higher social support reduces the impact of stress on pregnant women and decreases the risk of prenatal depressive symptoms [11]. The support provider can be either a family member, partner, friend, or another person or institution [11,12,13]. Partner and family support can be a major source of social support for pregnant women. Spousal support could also affect the psychological health of mothers [14,15]. Additional support can also come from childbirth classes. Attending childbirth classes is associated with an improvement in the emotional state of pregnant women [2].

Many studies indicate that adequately selected physical exercises and physical activity for pregnant women have an impact on the level of stress and anxiety and the occurrence of symptoms of depression during pregnancy, as well as reducing the risk of postpartum depression [16,17,18,19,20,21].

The WHO and the American College of Obstetricians and Gynecologists recommend 150 min of aerobic, moderate exercise per week both during pregnancy and after delivery. In addition, women who, before pregnancy, performed high-intensity aerobic exercise or were physically active daily may continue these activities during pregnancy and the postpartum period [18,22].

Hence, both social support in the form of a loved one or participation in childbirth classes, as well as physical activity, may have a protective effect on the emotional state of pregnant women. However, further research is needed to confirm the benefits of physical activity for pregnant women. Early prevention and intervention to reduce anxiety and perceived stress in pregnancy could hold the key to more advantageous early postnatal parenting [23].

Therefore, the aim of the study was to assess the level of perceived stress and anxiety in pregnant women depending on their physical activity and to answer the following questions: What was the level of perceived stress and anxiety in pregnant women depending on their physical activity both before and during pregnancy? What was the level of perceived stress and anxiety of the surveyed women depending on their marital status and participation in childbirth classes? What kind of factors affected the level of perceived stress?

It was assumed that physically active women before pregnancy and physically active women during pregnancy, women in a relationship, and women participating in childbirth classes are characterized by a lower level of perceived stress and anxiety.

## 2. Materials and Methods

### 2.1. Studied Group

The study was carried out between 2018 and 2020 at the Department of Gynecology and Obstetrics of the Clinical University Hospital in Wroclaw with the consent of the head of the department and with the consent of the Bioethics Committee of the Wroclaw University of Health and Sport Sciences, Poland (reference no. 40/2018). The study was conducted following the Helsinki Declaration.

The study involved 478 pregnant women reporting for checkups to their gynecologists. In Poland, pregnant and postpartum women (having Polish citizenship and residing in Poland) have the right to free prenatal care (preventive, diagnostic, and treatment). In accordance with the applicable and current Polish standards, the date of the visit for a woman is determined by gynecologist: once a month between 4 and 28 weeks of pregnancy, twice a month between 28 and 36 weeks, and every week from 36 weeks until delivery. Every visit is recorded in the “Documentation of Pregnancy”. The gynecologist informs the pregnant woman to report regularly and regardless of her well-being. Women in their 21st week of pregnancy can also benefit from free antenatal education conducted by midwives. However, the most popular in Poland are childbirth classes. According to the standards, free childbirth schools should operate at all units that run obstetric wards and care for pregnant women. Participation in these classes, as well as visits to the gynecologist, are not obligatory.

**Figure 1:**
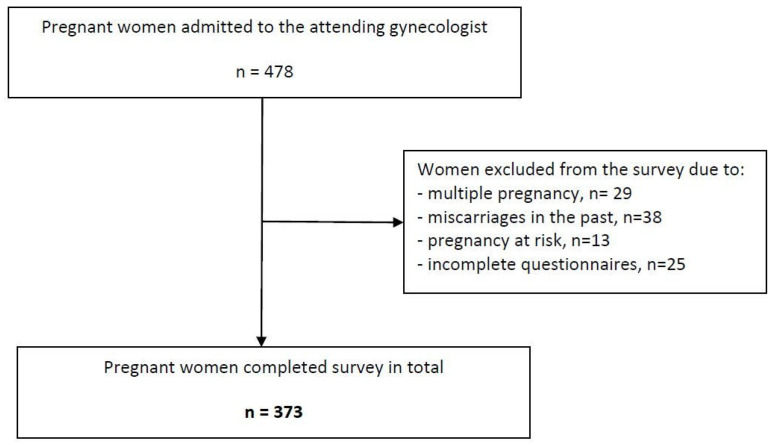
Recruitment process for the study sample.

The data from 373 women (Figure 1) who correctly completed the questionnaires and met the inclusion criteria: informed consent to participate in the study, primiparas, women with a singleton pregnancy, an ordinary course of pregnancy, being at the age of ≥18 years, having a gestational age of >20 weeks, no communication difficulties, or a mental deficiency, were qualified for the final statistical analysis.

Exclusion criteria were previous childbirths and miscarriages and having a diagnosis of a psychiatric disease (e.g., depression or/and anxiety disorders). Characteristics of the study sample are presented in Table 1.

### 2.2. Measurement Tools

The Polish adaptations of the Perceived Stress Scale (PSS-10), the State-Trait Anxiety Inventory (STAI), and a self-administered questionnaire were used. The self-administered questionnaire included basic socio-demographic data (i.e., age, education, marital status, type of employment) and questions related to physical activity undertaken, pregnancy, concerns, planned delivery, and attending childbirth classes.

The PSS-10, adapted in Polish by Juczyński and Ogińska-Bulik, includes 10 questions and assesses subjective feelings related to stressful situations. The examined person can obtain a maximum of 40 points. The higher the score, the higher the intensity of perceived stress. A score between 0 and 13 points indicates a low stress level, between 14 and 19 points an average stress level, and above 20 points indicates a high stress level. The Cronbach’s alpha was 0.86 [24].

The STAI, adapted in Polish by Sosnowski et al., evaluates anxiety as a state (STAI X-1) and anxiety as a trait (STAI X-2). Each of STAIs (STAI X-1 and STAI X-2) consists of 20 responses. The participant chooses one of four responses that describes how they feel at that particular moment (STAI X-1) and how they generally feel (STAI X-2). The higher the score, the higher the level of anxiety. The score dividing participants into groups with low and high levels of anxiety for the STAI X-1 was 44 points, and for the STAI X-2, 46 points. The Cronbach’s alpha was 0.89 for STAI X-1 and 0.83 for STAI X-2) [25].

### 2.3. Data Analysis

The following descriptive methods were used in the statistical analysis: mean, standard deviation, and, in the case of qualitative variables—numbers and percentages. The normality of the distribution was verified using the Shapiro-Wilk test. In order to compare two subgroups (i.e., active women before pregnancy vs. non active women before pregnancy; active women during pregnancy vs. non-active women during pregnancy; women attending childbirth classes vs. not attending childbirth classes; single vs. women in a relationship), the *t*-Student’s test for independent groups was used. To determine the quantity of the effect of differences between the examined groups, a Cohen’s d test was used. In order to identify factors affecting the PSS-10 scores, a multiple linear regression analysis was done. Statistical tests were verified at the significance level of *p* < 0.05.

## 3. Results

The mean level of perceived stress in the entire study group was 20.1 (±5.7), which corresponds to a high score. A high level of perceived stress was noted in as many as 244 (66%) women. A low level of stress was characteristic of 42 (11%) pregnant women.

The mean levels of anxiety as a state and as a trait were 46.4 (±6.3) and 46.7 (±6.4), respectively. A high level of anxiety as a state was noted in over 79% of the surveyed women (296 women), and a high level of anxiety as a trait in over 59% (221 women).

The surveyed women were most afraid of complications during childbirth (*n* = 281), pain (*n* = 218), and poor health of the child (*n* = 175).

To the question included in the self-administered questionnaire: “Do you feel stressed before childbirth?” nearly 78% of the pregnant women answered affirmatively. In this group, significantly higher values of anxiety as a trait and anxiety as a state were noted compared to women who responded to the question negatively (*p* = 0.0120 and *p* = 0.0008, respectively).

There were no statistically significant differences in the level of perceived stress and anxiety between the groups of physically active and inactive women during pregnancy.

However, the mean stress level was significantly lower among women who were physically active before pregnancy. There were no statistically significant differences in the level of anxiety between the groups of physically active and physically inactive women before pregnancy (Table 2).

**Figure 2:**
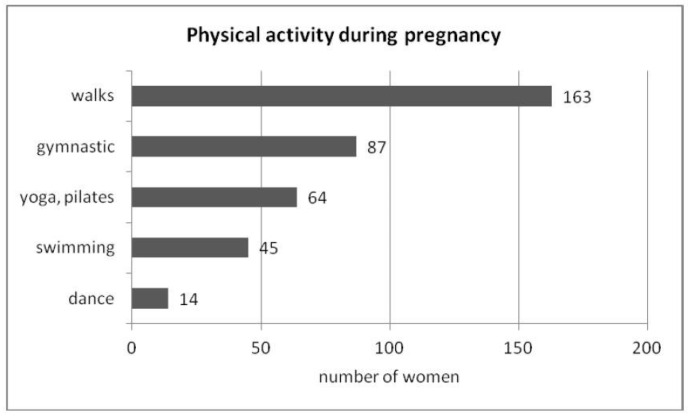
Type of physical activity during pregnancy.

Among the surveyed women, walking and gymnastics were the most frequently chosen forms of physical activity during pregnancy (Figure 2).

A comparative analysis showed a significantly higher level of perceived stress and anxiety as a state among single women compared to women in relationships.

In addition, women attending childbirth classes were characterized by significantly lower values of perceived stress and anxiety as a state and anxiety as a trait (Table 3).

A multiple linear regression analysis showed that there was a moderately significant effect between women’s age, marital status, week of pregnancy, physical activity before pregnancy, physical activity during pregnancy, attending childbirth classes, STAI (X-1), STAI (X-2), and PSS-10. From these independent variables, the following were not significant as predictors for PSS-10: marital status, week of pregnancy, physical activity during pregnancy, and STAI (X-1). Therefore, these variables were excluded from the model. The individual factors were examined further and indicated that women’s age, physical activity before pregnancy, participation in childbirth classes, and STAI (X-2) had the greatest impact on PSS-10 scores. These factors explain 30.1% of the PSS-10 scores (Table 4).

## 4. Discussion

The obtained results confirm the frequent occurrence of high stress and anxiety levels in pregnant women. In the study group of women, these results were lower in the case of stress (66%) and higher in the case of anxiety (79%) compared to the study by Tang et al. (91.86% and 15.04%, respectively) but significantly higher compared to studies by Obrochta et al. (nearly 11% and 20%, respectively) [1,26]. Similar results were reported in the studies by Jonsdottir et al. of 52% and 47%, respectively [15]. Many factors contribute to these discrepant results, including differences in the screening scales used, the week of pregnancy in which the tests were performed, and differences in culture or the obstetric system, which may directly influence expectations, feelings of control, and thus probably the level of anxiety and stress. The high level of perceived stress and anxiety also increases the fears that accompany women during pregnancy. Complications during childbirth, pain, and fear for the child’s health are most often mentioned [2,17]. In particular, this applies to primiparas with higher expectations of labour pain, making them more prone to anxiety [1]. Nevertheless, as the authors emphasize, high levels of stress and anxiety are common and severe problems that may affect the delivery itself, the postpartum period, and the health of the mother and child [9,23]. Prevention and rapid interventions early in pregnancy can reduce this level. The protective factors include social support and physical activity [1,2].

Meanwhile, the obtained results did not show a significant difference in the level of stress and anxiety in the groups of women depending on the physical activity undertaken during pregnancy. However, many studies indicate that physical exercise during pregnancy is safe for pregnant women and can have multiple health benefits [19,20,21]. Among other things, it can reduce stress and anxiety levels and the symptoms of depression. It can also calm women by changing physical mechanisms such as hormone secretion [1,2,18,27]. Other researchers also report a positive impact of activities such as walking and yoga during pregnancy on women’s stress, anxiety, and mood levels [28,29]. Even 30 min of walking three times a week can significantly minimize depressive symptoms in pregnant women [18]. It should be emphasized that physical activity during pregnancy can have a long-term impact on women’s emotional states. The long-term impact of physical activity during pregnancy was also reported by other researchers [2,16,17,30].

On the other hand, women who were physically active before pregnancy were characterized by a significantly lower level of stress during pregnancy. A similar trend was noted for anxiety as a state. This confirms that regular physical activity and an active lifestyle have long-term benefits [20]. Kołomańska-Bogucka et al. reported that physically active women (before and/or during pregnancy) had a lower risk of developing depression than non-active women. Physical activity also had an impact on reducing both the level of anxiety and stress [18].

In addition, it was shown that social support (in the case of the presented studies, it was the presence of a partner and being in a relationship, as well as participation in childbirth classes) is significantly related to the level of perceived stress and anxiety experienced by pregnant women. Single women were characterized by substantially higher levels of perceived stress and anxiety than those in relationships. These results are consistent with the reports of other researchers, who emphasize that pregnant women who lack support from partners and have poor relationships with their husbands are more likely to have prenatal depression. In addition, they indicate that there is a relationship between the occurrence of depression and fear of childbirth, weak marital bonds, and a lack of social support, increasing the risk of postpartum depression [1,2,31]. Women who were not satisfied with their relationships were four times more likely to experience perinatal distress [15]. Higher social support reduces the impact of stress on pregnant women, decreasing the risk of prenatal depressive symptoms [11], and a lack of social support is a risk factor for depression during pregnancy [32]. Other researchers found that, as part of social support, the family plays the most critical role in reducing maternal stress [33] and can help women maintain good mental health during pregnancy [1]. This suggests that we can help increase the level of social support for pregnant women by preparing their spouses or partners (e.g., education, encouraging them to attend childbirth classes). Therefore, childbirth classes should prepare not only women but also their spouses or partners for childbirth and influence their perceived stress and anxiety levels. Obtained results showed that women participating in childbirth classes had a significantly lower level of perceived stress and anxiety than women who did not attend such classes.

Regression analysis also confirmed this. Among the selected factors, participation in childbirth classes, physical activity before pregnancy, the level of anxiety as a trait, and age had the greatest impact on the stress level of the surveyed women. It seems that the presence of anxiety among these factors is evident because stress is an important risk factor for anxiety in early pregnancy [11], and anxiety symptoms are also a risk factor for early pregnancy stress [34]. There is a strong association between stress and anxiety.

The risk of pregnancy complications and concerns related to pregnancy and childbirth increases with age, which can affect the level of stress and anxiety [2]. Most researchers, however, emphasize that young age is a factor in pregnancy anxiety [35], although this can also be related to lower socioeconomic status, teenage pregnancy, lack of education, unplanned or unwanted pregnancy, etc.

However, it is worth keeping in mind that the other two factors are modifiable. This suggests that we can help decrease the level of stress and anxiety in pregnant women. The study results also suggest that physical activity may be an important factor in preventing prenatal mental disorders. Physical activity during pregnancy was very important, but physical activity before pregnancy was more protective and important. In addition, participation in childbirth classes together with a partner/husband can be a source of valuable support for both the pregnant woman and her partner/husband, as well as an opportunity to start regular physical activity. McCarthy et al. emphasize that women’s social support and the provision of clear and consistent information are also essential to minimizing stress and anxiety in the perinatal period [6]. This is also confirmed by Kowalska et al., who found that after 10 weeks in childbirth classes, the stress level decreased in the group of women who were active before pregnancy compared to women who were not physically active before pregnancy [2].

Therefore, the benefits of prenatal physical activity in reducing stress and anxiety during pregnancy should be disseminated. Given the results, it seems worthwhile to stimulate women to remain physically active during pregnancy and additionally encourage them to participate in childbirth classes. Most importantly, however, regular physical activity should be promoted among women who plan to have a family.

It seems indisputable that emotional states must be monitored at every stage of pregnancy and in the postpartum period in order to introduce interventions as soon as possible. Interventions implemented during pregnancy may reduce the risk of postpartum psychological distress [26].

### Limitations

The present study had some limitations. The tests used were screening in nature and were not related to the diagnosis (e.g., anxiety disorders). Biochemical stress factors, e.g., cortisol levels, have not been studied. In the case of the presented studies, social support meant only being in a relationship or participating in childbirth classes. No scales were used. Additionally, physical activity was noted only in the self-questionnaire. It was a single-centre study and the tests were performed in one hospital. Despite the same standards applicable in Polish prenatal care in hospitals, the results need to be interpreted with caution for other populations. Moreover, the study was carried out at a single point in time, which is a clear limitation as it does not show the dynamics of changes in the emotional state of the pregnant women. The study took into account only a few selected factors that may affect the level of stress and anxiety. Further research among pregnant women and women in the postpartum period is needed to confirm the short-term and long-term benefits of physical activity and identify as many factors as possible that may affect the emotional state of women. More knowledge is needed to identify factors that influence the level of perceived stress and anxiety during pregnancy.

## 5. Conclusions

In the group of pregnant women, a high level of perceived stress and anxiety as a state and as a trait were noted. Physically active women before pregnancy, women attending childbirth classes and remaining in a relationship were characterized by a lower level of perceived stress and anxiety compared to physically inactive women or women who did not participate in childbirth classes and were single.

Participation in childbirth classes, physical activity before pregnancy, the level of anxiety as a trait, and women’s age had the most significant impact on the level of perceived stress in the group of pregnant women.

## Figures and Tables

**Table 1:** Characteristics of the study sample, N = 373.

Feature	Mean (SD) or N (%)
Age [years]	29.7 (4.1)
Range	18–47
Education	
Primary and vocational	56 (15)
Secondary and higher	317 (85)
Type of employment	
Employment contract	317 (85.0)
Contract of mandate	16 (4.3)
Unemployed	8 (2.1)
Other	32 (8.6)
Marital status	
Single In a relationship (cohabitation or marriage)	82 (22.0) 291 (78.0)
Place of residence	
Urban	324 (86.9)
Non-Urban	49 (13.1)
Marriage lenght [years]	3.3 (2.5)
Range	0.5–13
Week of pregnancy	28.6 (4.1)
Range	21–41
Physical activity before pregnancy	
Yes	267 (71.6)
No	106 (28.4)
Physical activity during pregnancy	
Yes	215 (57.6)
No	158 (42.4)
Perceived stress before childbirth	
Yes	291 (78.0)
No	82 (22.0)
Childbirth classes	
Yes	301 (80.7)
No	72 (19.3)
Planned family birth (birth with close relative/husband/partner)	
Yes	222 (60.0)
No/I do not know	151(40.0)

**Table 2:** The studied parameters depend on physical activity during pregnancy and physical activity before pregnancy (*t*-Student test for independent groups).

Scales	Physical Activity during Pregnancy								
	Yes			No			Test *t*-Student		Effect Size
	*n*	Mean	SD	*n*	Mean	SD	*t*	*p*	Cohen’s d
PSS-10	215	19.9	5.2	158	20.4	6.3	−0.7	0.4552	0.0865
STAI (X-1)	215	46.6	6.0	158	46.2	6.6	0.6	0.5291	0.0634
STAI (X-2)	215	46.9	6.4	158	46.3	6.5	0.9	0.3552	0.0930
	Physical Activity before Pregnancy								
	Yes			No			Test *t*-Student		Effect Size
	*n*	Mean	SD	*n*	Mean	SD	*t*	*p*	Cohen’s d
PSS-10	267	19.8	5.5	106	21.1	6.2	−1.9	0.0472 *	0.2218
STAI (X-1)	267	46.3	6.5	106	46.7	5.6	−0.6	0.5590	0.0659
STAI (X-2)	267	46.8	6.7	106	46.4	5.7	0.5	0.5972	0.0643

PSS-10, Perceived Stress Scale; STAI X-1, State-Trait Anxiety Inventory as a state; STAI X-2, State-Trait Anxiety Inventory as a trait; * *p* < 0.05.

**Table 3:** The studied parameters depend on participation in the childbirth classes (*t*-Student test for independent groups).

	Participation in the Childbirth Classes								
	Yes			No			Test *t*-Student		Effect Size
	*n*	Mean	SD	*n*	Mean	SD	*t*	*p*	Cohen’s d
PSS-10	301	19.7	6.0	72	22.1	3.6	3.4	0.0009 *	0.4851
STAI (X-1)	301	44.8	5.6	72	53.2	2.8	12.3	0.00001 *	1.8973
STAI (X-2)	301	44.6	5.1	72	55.3	3.7	17.0	0.00001 *	2.4016
	Marital Status								
	Single			In relationship			*t*	*p*	Cohen’s d
PSS-10	82	21.9	3.6	291	19.6	6.1	3.2	0.0007 *	0.4539
STAI (X-1)	82	47.7	4.2	291	46.0	6.7	2.1	0.0175 *	0.2959
STAI (X-2)	82	46.7	4.6	291	46.6	6.9	0.05	0.1784	0.1784

PSS-10, Perceived Stress Scale; STAI X-1, State-Trait Anxiety Inventory as a state; STAI X-2, State-Trait Anxiety Inventory as a trait; * *p* < 0.05.

**Table 4:** Multiple linear regression analysis exploring the effects of the selected factors on PSS-10 scores.

	Coefficient Value	±95% CI	*p*
Age	0.17	0.05–0.29	0.0050 *
Physical activity before pregnant	1.43	0.34–2.52	0.0101 *
Attending to childbirth classes	−1.63	−2.59–0.67	<0.00001 *
STAI (X-2)	0.44	0.36–0.51	<0.00001 *
R^2^	0.301		
R^2^_adj_	0.29		
Effect size	0.39		

STAI X-2, State-Trait Anxiety Inventory as a trait; * *p* < 0.05.

## Data Availability

The data presented in this study are available on request from the corresponding author.
